# Pre-Clinical Evaluation of the Hypomethylating Agent Decitabine for the Treatment of T-Cell Lymphoblastic Lymphoma

**DOI:** 10.3390/cancers15030647

**Published:** 2023-01-20

**Authors:** Lien Provez, Tom Putteman, Mattias Landfors, Juliette Roels, Lindy Reunes, Sara T’Sas, Wouter Van Loocke, Béatrice Lintermans, Stien De Coninck, Morgan Thenoz, Wouter Sleeckx, Natalia Maćkowska-Maślak, Tom Taghon, Marc R. Mansour, Nadine Farah, Koen Norga, Peter Vandenberghe, Rishi S. Kotecha, Steven Goossens, Sofie Degerman, Renate De Smedt, Pieter Van Vlierberghe

**Affiliations:** 1 Normal and Malignant Hematopoiesis Laboratory, Department of Biomolecular Medicine, Ghent University, 9000 Ghent, Belgium; 2 Cancer Research Institute Ghent (CRIG), 9000 Ghent, Belgium; 3 Taghon Laboratory, Department of Diagnostic Sciences, Ghent University, 9000 Ghent, Belgium; 4 Department of Medical Biosciences, Umeå University, 90185 Umeå, Sweden; 5 Unit for Translational Research in Oncology, Department of Diagnostic Sciences, Ghent University, 9000 Ghent, Belgium; 6 Hematology Department, Ghent University Hospital (UZGent), 9000 Ghent, Belgium; 7 Institute of Human Genetics, Polish Academy of Sciences, 60-479 Poznań, Poland; 8 Leukaemia Biology Research Group, Department of Heamatology, University College London Cancer Institute, London WC1E 6DD, UK; 9 UCL Great Ormond Street Institute of Child Health, London WC1N 1EH, UK; 10 Paediatric Oncology at Antwerp University, 2000 Antwerp, Belgium; 11 Department of Human Genetics, Leuven University, 3000 Leuven, Belgium; 12 Leukaemia Translational Research Laboratory, Telethon Kids Cancer Centre, Telethon Kids Institute, University of Western Australia, Perth 6009, Australia; 13 Department of Clinical Haematology, Oncology, Blood and Marrow Transplantation, Perth Children’s Hospital, Perth 6009, Australia; 14 Curtin Medical School, Curtin University, Perth 6102, Australia; 15 Department of Clinical Microbiology, Umeå University, 90185 Umeå, Sweden

**Keywords:** T-LBL, decitabine, DNA methylation

## Abstract

**Simple Summary:**

T-cell lymphoblastic lymphoma (T-LBL) is a rare and aggressive lymphatic cancer, most often diagnosed in teenagers and young adults. Nowadays, patients are treated with chemotherapy or undergo a hematopoietic stem cell transplantation. This can cause many short- and long-term side-effects and relapse still occurs. Therefore, finding less toxic anti-lymphoma therapies is hoped for. One epidrug of interest is decitabine, a DNA hypomethylating agent that targets the aberrant DNA methylation profile, which previously was FDA-approved for AML and MDS. With the results in this manuscript, we especially hope to provide pre-clinical proof that is necessary to include decitabine as a therapeutic agent in clinical trials for the treatment of T-LBL. Moreover, with the reported downstream effects of decitabine treatment, we hope to further increase the understanding of the working mechanisms of decitabine, which could ultimately result in better treatment protocols and associated biomarkers for T-LBL patients.

**Abstract:**

T-cell lymphoblastic lymphoma (T-LBL) is a rare and aggressive lymphatic cancer, often diagnosed at a young age. Patients are treated with intensive chemotherapy, potentially followed by a hematopoietic stem cell transplantation. Although prognosis of T-LBL has improved with intensified treatment protocols, they are associated with side effects and 10–20% of patients still die from relapsed or refractory disease. Given this, the search toward less toxic anti-lymphoma therapies is ongoing. Here, we targeted the recently described DNA hypermethylated profile in T-LBL with the DNA hypomethylating agent decitabine. We evaluated the anti-lymphoma properties and downstream effects of decitabine, using patient derived xenograft (PDX) models. Decitabine treatment resulted in prolonged lymphoma-free survival in all T-LBL PDX models, which was associated with downregulation of the oncogenic MYC pathway. However, some PDX models showed more benefit of decitabine treatment compared to others. In more sensitive models, differentially methylated CpG regions resulted in more differentially expressed genes in open chromatin regions. This resulted in stronger downregulation of cell cycle genes and upregulation of immune response activating transcripts. Finally, we suggest a gene signature for high decitabine sensitivity in T-LBL. Altogether, we here delivered pre-clinical proof of the potential use of decitabine as a new therapeutic agent in T-LBL.

## 1. Introduction

T-cell lymphoblastic lymphoma (T-LBL) is an aggressive hematological cancer characterized by the uncontrolled proliferation of progenitor T-cells. Although T-LBL patients show lower infiltration of cancer cells into the bone marrow (<25%) as compared to patients with T-cell acute lymphoblastic leukemia (T-ALL) (>25%), T-LBL and T-ALL are considered the same disease according to the World Health Organization since they have similar immunophenotypic and morphological features. Due to a lack of new therapeutic strategies for T-LBL, and the similarities between T-LBL and T-ALL, patients with T-LBL are currently treated with T-ALL-designed protocols [1], comprising intensified chemotherapy and potentially also an allogeneic hematopoietic stem cell transplantation (HSCT). Survival rates reach 80%, but current therapies cause many short- and long-term side effects, such as a low blood cell count, infections, graft versus host disease and Tumor Lysis Syndrome. Additionally, relapse leading to a dismal prognosis can still occur [2]. Therefore, it is essential to continue looking for more effective and less toxic therapies.

**Figure 1:**
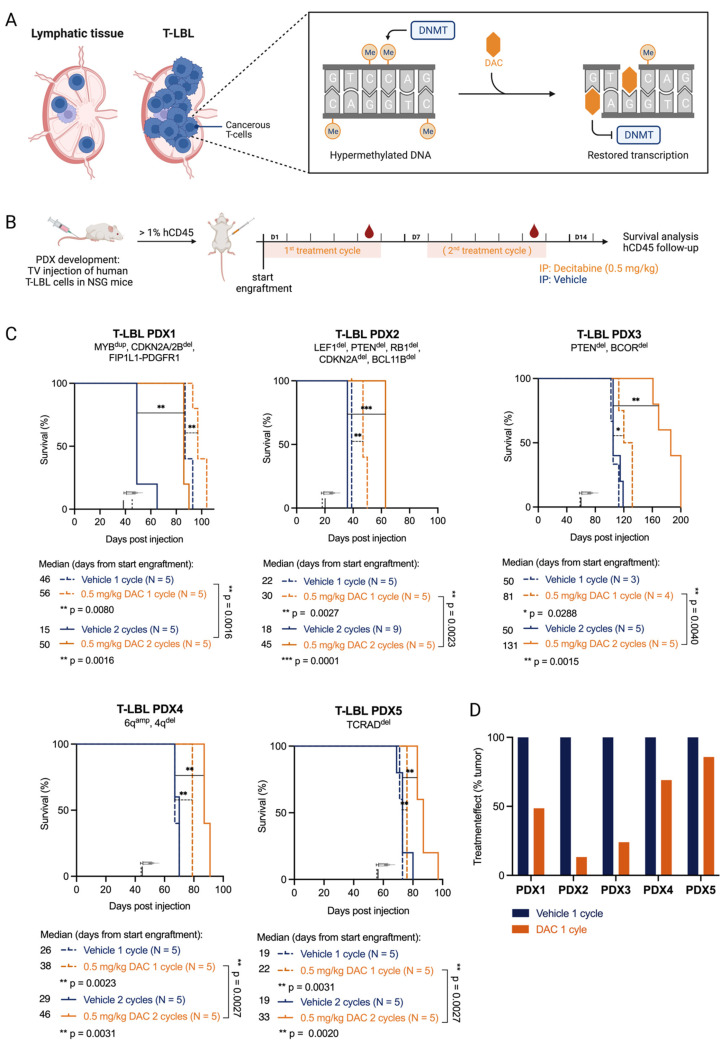
T-LBL PDX models are sensitive towards in vivo treatment with decitabine. (**A**) T-LBL develops in lymphatic tissues such as the lymph nodes. In T-LBL cells, the DNA methylation pattern is hypermethylated compared to healthy cells. Upon replication, decitabine (DAC) can incorporate into the DNA and irreversibly bind to DNA methyl transferases (DNMTs), thereby countering hypermethylation and restoring transcription. Figure created with BioRender.com. (**B**) NSG mice injected with malignant pleural effusions from patients with T-LBL were treated with vehicle (PBS with 1% DMSO, blue) or decitabine (DAC 0.5 mg/kg body weight, orange). Once engraftment was successful (1% hCD45^+^ cells in the peripheral blood), mice were treated for one or two cycles for five consecutive treatment days followed by two days off. Mice were followed for survival analysis and leukemia burden in the blood. Figure created with BioRender.com. (**C**) Kaplan–Meier analysis of lymphoma-free survival after decitabine treatment (start of treatment is marked with a syringe) is shown (log-rank Mantel-Cox test; *p*-values: 0.1234 (ns), 0.0332 (*), 0.0021 (**), 0.0002 (***)) for the five T-LBL PDX models analyzed. One treatment cycle (dotted lines) and two treatment cycles (full lines) are compared. (**D**) The treatment effect of 1 cycle of decitabine on hCD45^+^ cells in the peripheral blood is shown by normalizing tumor growth (average #hCD45^+^ cells on day 5/average #hCD45^+^ cells on day 1) in decitabine treated mice to tumor growth in vehicle treated mice.

One of the hallmarks of human cancer is aberrantly methylated DNA profiles [3]. For T-LBL and T-ALL, a hypermethylated DNA profile has been identified in a subset of cancer patients [4,5]. Targeting this aberrant DNA methylation profile using DNA hypomethylating agents is a promising therapeutic strategy. The DNA hypomethylating agent azacytidine and its deoxy derivative decitabine are clinically used for the treatment of acute myeloid leukemia (AML) and myelodysplastic syndrome (MDS) [6]. These two cytidine analogues are incorporated into the DNA strand upon replication. They irreversibly bind to DNA methyltransferases (DNMT), trapping the enzyme in a covalent manner and, as such, inducing global DNA hypomethylation (Figure 1A). Whereas decitabine is only able to incorporate into DNA, about 85% of azacytidine also incorporates into the RNA [7]. This might explain differences in drug efficacy and toxicity between azacytidine and decitabine. Indeed, decitabine has been reported to show higher efficacy in elderly AML patients and low-risk MDS patients, while azacytidine seems to be a better choice for high-risk MDS patients [8,9,10].

Recently, azacytidine has been shown to have anti-leukemic effects in the hypermethylated T-ALL subtypes but not in the hypomethylated T-ALL subtypes, while decitabine demonstrated a therapeutic response in all T-ALL subtypes tested [5,11]. Until now, neither azacytidine nor decitabine have been studied for therapeutic use in T-LBL other than in a single case study where decitabine combined with the CAG regimen (low-dose cytarabine, aclarubicin hydrochloride and granulocyte colony-stimulating factor) was shown to exert an effect in one patient with T-LBL [12]. We have therefore built on this knowledge to characterize the effect of decitabine in T-LBL, providing the preclinical evidence that is required to include decitabine in clinical trials for T-LBL.

## 2. Materials and Methods

### 2.1. Patient Characterization

The medical records, including age at diagnosis and sex, of the patients with T-LBL were received together with the primary patient material. Whole exome sequencing was performed (Macrogen, Seoul, Republic of Korea) on the Illumina NovaSeq6000 platform (Illumina, Inc., San Diego, CA, USA) using primary patient samples from pleural effusion and not infiltrated control bone marrow from the same patient. Copy number variation sequencing was performed on genomic DNA using 200 bp shearing for healthy patient material, cancerous patient material and PDX material. Immunophenotyping was performed in a clinical setting using the EuroFlow panel [13]. Gene expression profiling and DNA methylation profiling were performed as mentioned below.

### 2.2. In Vivo Treatment of Xenografts

Primary T-LBL patient samples for establishing patient-derived xenograft (PDX) models were acquired by informed consent through the Department of Pediatric Hematology-Oncology at Ghent University Hospital, Leuven University Hospital, Antwerp University, University College London and Perth Children’s Hospital.

Male NOD/SCIDγ (NSG) mice were injected with malignant pleural effusion samples from patients with T-LBL via the tail vein for the development of the PDXs. For all our experiments, second generation PDX mice were used, starting from cryovials of the primary xenografts. For each PDX model, the same graft material was used for all experiments. Lymphoma engraftment was regularly monitored by flow cytometry, using human CD45^+^ (hCD45) staining (CD45-FITC antibody (Miltenyi Biotec, Bergish Gladbag, Germany)). To correct for lymphoid depletion induced by the treatment, Precision Count Beads (BioLegend, San Diego, CA, USA) were added to a fixed volume of peripheral blood. An exact number of hCD45^+^ cells per volume of peripheral blood (30 μL) was calculated using:

(1) $$\mathrm{Absolute}\;\mathrm{Cell}\;\mathrm{Count}\;\left(\mathrm{cells}/\mu L\right)=\frac{{\mathrm{Cell}\;\mathrm{Count}\;\times \;\mathrm{Precision}\;\mathrm{Count}\;\mathrm{Beads}}^{\mathrm{TM}}\;\mathrm{Volume}}{{\mathrm{Precision}\;\mathrm{Count}\;\mathrm{Beads}}^{\mathrm{TM}}\;\mathrm{Count}\;\times \;\mathrm{Cell}\;\mathrm{Volume}}{\;\times \;\mathrm{Precision}\;\mathrm{Count}\;\mathrm{Beads}}^{\mathrm{TM}}\;\mathrm{Concentration}\;$$

For survival experiments, mice were treated via intraperitoneal injection (IP) with vehicle (PBS with 1% DMSO) or decitabine (0.5 mg/kg bodyweight, A3656 (Merck Life Science, Darmstadt, Germany)) for one or two cycles (5 days on, 2 days off) once 1% of leukocytes were hCD45^+^ to ensure all mice lived until the end of the treatment. Mice were randomly divided into groups of 5 PDX mice per condition.

For RNA and DNA collection, mice were treated via IP with vehicle or decitabine (0.5 mg/kg bodyweight) for 5 days once 50% of leukocytes were hCD45^+^ to ensure a sufficiently large hCD45^+^ population at the end of the experiment. In each group, 4 PDX mice were included. On day 8, mice were sacrificed and after red blood cell lysis, tumor cells were collected from the spleen (PDX1-4) or bone marrow (PDX5), depending on the location of the leukemic blasts.

All in vivo experiments were approved by the ethical committee of laboratory animal experimentation of the Faculty of Medicine and Health Sciences of Ghent University, under the ethic code ECD20-68.

### 2.3. DNA Methylation Profiling

DNA from full spleen or hCD45^+^ sorted bone marrow was isolated using the QIAamp DNA Mini Kit (Qiagen, Hilden, Germany). For each PDX model, biological triplicates or quadruplets were analyzed by GenomeScan (Leiden, The Netherlands) using the Infinium MethylationEPIC arrays (Illumina, Inc., San Diego, CA, USA). β-values, as defined in Illuminas Genomestudio software, were extracted using the minfi package in R [14] and normalized for bead type using BMIQ [15]. CpGs with detection *p*-value > 0.05 were considered missing and were imputed (K-nearest Neighbors) for the calculation of epigenetic age. Multimapping probes and probes with SNPs within 5 bp [16], as well as meQTLs [17], were removed from further analysis.

### 2.4. Gene Expression Profiling

RNA from full spleen or hCD45^+^ sorted bone marrow was isolated using the RNeasy Plus Kit (Qiagen, Hilden, Germany). Library preparation was performed by NXTGNT (Ghent, Belgium) using QuantSeq 3′mRNA-Seq FWD for Illumina (Lexogen GmbH, Vienna, Austria). For each PDX model, biological triplicates or quadruplets were analyzed by polyA RNA sequencing on the Illumina NextSeq500. Reads were aligned to GRCh38 using STAR2.7.6a. RNA sequencing counts were analyzed using the DESeq2 packages in R and genes with adjusted *p*-values < 0.05 were considered differentially expressed. Gene annotations include both coding and non-coding genes. Heatmaps were generated using the pheatmap package. Volcano plots were generated with the ggplot package. Chromosome X and Y were included in the analysis. These gene expression profiles were used to determine the T-LBL subtypes of the PDX models.

### 2.5. Data Analysis

GraphPad Prism 9.0 (GraphPad Software, San Diego, CA, USA) was used for statistical analysis of survival using the log-rank Mantel Cox test and for visualization of Kaplan–Meier curves, leukemic burden and EpiTOC Mitotic age.

T-LBL models were classified according to their CIMP subtype [18]. The heatmap was made in R using Euclidian distance matrix and complete hierarchical clustering. CIMP methylation percentage was calculated as previously described [19]. The EpiTOC Mitotic age was calculated as a mean beta value of all the EpiTOC CpGs present on the epic array [20]. Preranked gene set enrichment analyses with the hallmarks gene sets collection were performed in the GSEA desktop application (version 4.2.3) for each PDX model individually or all samples taken together.

To integrate DNA methylation and gene expression analysis, CpGs were considered differentially methylated if the absolute Δβ was greater than 0.2. HG38 annotation for the methylation array was used and matched to RNA sequencing data [16]. The locations of the Illumina cg probes were determined using UCSC Genome Browser.

### 2.6. HiC Analysis

Active and inactive compartments in T-ALL were defined based on previously published HiC data [21]. Data were retrieved from GEO for four T-ALL samples (GSM3967118, GSM3967119, GSM3967120 and GSM3967121). Reads were trimmed using homerTools trim with options-GATC-mis 0-matchStart 20-min 20. Next, Bowtie2 was used for alignment using the GRCh38 reference genome. The Homer suite was used for further processing the HiC data using the functions makeTagDirectory; analyzeHiC. The function runHiCpca.pl. with a resolution of 50,000 was used to determine active and inactive compartments. To create a pan-T-ALL map of active and inactive regions, compartments were called based on consensus A/B calls for each T-ALL individually. Discordant calls were marked as ambiguous and not further used in the analysis. To correlate the differentially methylated CpGs and differentially expressed genes with open and closed regions of the chromosome, the HiC status determined for each gene was included in the analysis. To calculate the proportions of CIMP CpGs in the open and closed chromatin, genes with a HiC status in A or B regions were included, while genes in the A_B region or with no info were excluded. The Circos figures were made using RCircos package in R.

## 3. Results

### 3.1. T-LBL PDX Models Are Sensitive to the FDA-Approved Hypomethylating Agent Decitabine

To study the therapeutic value of DNA hypomethylating agents in T-LBL, we evaluated the anti-lymphoma effects of decitabine in a preclinical setting using five human T-LBL patient-derived xenograft (PDX) models. All five PDX models were extensively characterized using exome sequencing, copy number variation analysis, RNA sequencing, immunophenotyping and DNA methylation profiling (Supplementary File S1). Genetically, the five different PDX models belong to different genetic subtypes: *TLX3*^+^ (PDX1), *NKX2-5*^+^ (PDX2), *NKX2-1*^+^ (PDX3), *HOXA*^+^ (PDX4) and *TAL/LMO*^+^ (PDX5). We also determined the CIMP subtype of the PDX models using a CpG island methylator phenotype panel (>40% methylated CIMP CpGs = CIMP^+^, <40% methylated CIMP CpGs = CIMP^−^) which has previously been shown to have prognostic relevance in T-ALL [4,5,19]. DNA methylation classification has been used as a biomarker with regards to drug sensitivity for DNA hypomethylating agents [4,5,22] and as a prognostic marker for outcomes in a wide range of malignancies including T-ALL/T-LBL, colorectal cancer and glioma [4,5,22,23,24]. PDX1 (89.4% CIMP methylation percentage), PDX3 (95.2%) and PDX4 (89.7%) showed CIMP hypermethylation and were CIMP^+^, whereas PDX2 (43.3%) and PDX5 (53.0%) showed rather intermediate CIMP methylation but were, by definition, still CIMP^+^ [4]. Since CIMP subtypes were previously associated with proliferative history in T-ALL [5], we predicted the mitotic age of our T-LBL models using the Epigenetic Timer of Cancer (EpiTOC) [20]. Additionally, in T-LBL samples, we identified a correlation between the EpiTOC biological age of the PDX models and the percentage of methylated CIMP CpGs in the CIMP panel, with lower methylation percentages having a shorter history of proliferation in comparison with higher methylation percentages (Supplementary Figure S1).

To evaluate the therapeutic potential of decitabine in vivo, we treated all five T-LBL PDX models with either one or two cycles (5 days on, 2 days off, 0.5 mg/kg via daily intraperitoneal injections) of decitabine (Figure 1B). All PDX models showed significantly prolonged survival upon decitabine treatment (Figure 1C). When we compared PDX models treated for one versus two cycles of therapy, all PDX models showed extended survival after two cycles (Figure 1C). The benefit of two treatment cycles of decitabine compared to vehicle was most pronounced in three out of five PDX models (PDX1, PDX2, PDX3), which survived twice as long compared to the vehicle group, whereas a significant but less pronounced benefit of decitabine treatment was seen for PDX4 and PDX5. The efficacy of the treatment was also correlated with the drop in absolute numbers of hCD45^+^ lymphoblasts in the peripheral blood, as documented by flow cytometry (Figure 1D, Supplementary Figure S2). Based on similar treatment effects on both survival and numbers of hCD45^+^ cells, we identified a more sensitive subgroup (PDX1, PDX2, PDX3) and a less sensitive subgroup (PDX4 and PDX5) towards decitabine treatment.

In addition, all mice treated with decitabine also showed leukocyte depletion during the treatment, with a significant reduction in non-leukemic mCD45^+^ cells in the peripheral blood. Once treatment was finished, the restoration of the leukocyte population occurred within five days (Supplementary Figure S3A). While tracking leukemia burden using hCD45^+^ cells, precision count beads were used to correct for this depletion (Supplementary Figure S3B). Body weight loss of more than 20% occurred in two out of seven PDX2 mice treated with two cycles of decitabine. Those mice were excluded from the analysis. No other toxicities were observed.

Overall, these results show that decitabine prolonged survival in all five T-LBL PDX models and therefore represents a promising option for the treatment of patients with T-LBL.

### 3.2. Overall Anti-Lymphoma Effects of Decitabine Treatment Are Mediated by Downregulation of the MYC Pathway

**Figure 2:**
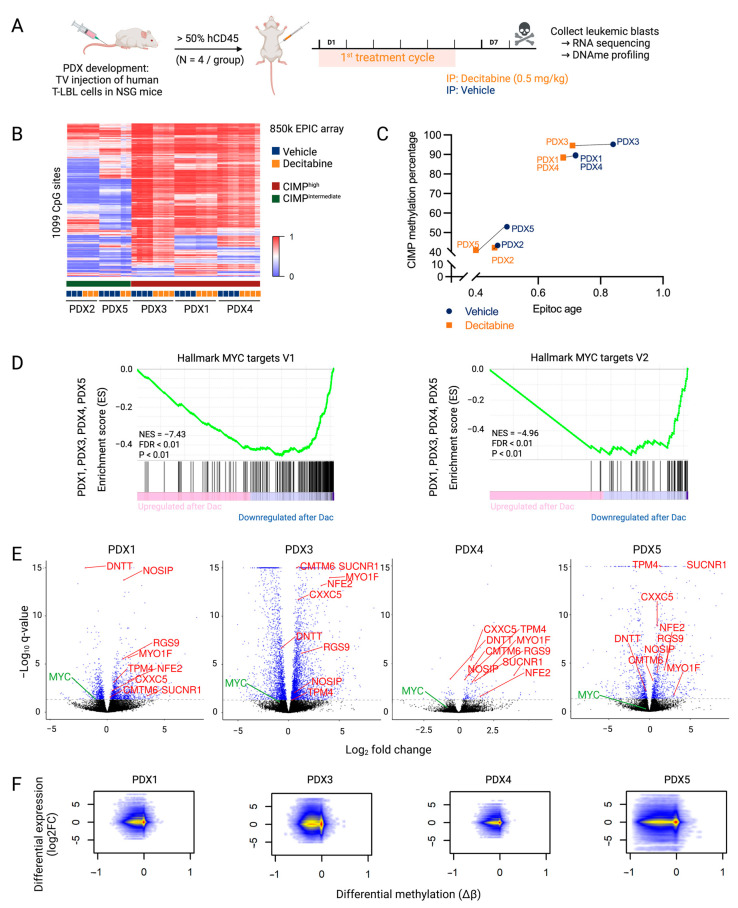
Downstream effects of decitabine treatment that mediate anti-lymphoma effects in T-LBL. (**A**) NSG mice were engrafted with malignant pleural effusion material of T-LBL patients. Once engraftment percentage in the blood was higher than 50%, mice were treated with vehicle (PBS with 1% DMSO, blue) or decitabine (0.5 mg/kg body weight, orange) for one cycle comprising of five consecutive treatment days followed by two days off. Mice were sacrificed on day eight for the collection of leukemic blasts. Figure created with BioRender.com. (**B**) β-values of CIMP CpG in the five T-LBL PDX models after decitabine or vehicle treatment. Methylation at these 1099 CpG sites was used to determine CIMP T-LBL subtypes. (**C**) Correlation between EpiTOC age and the CIMP methylation percentage paired per PDX model after decitabine or vehicle treatment. (**D**) Preranked GSEA from PDX1, PDX3, PDX4 and PDX5 targets in decitabine versus vehicle conditions showing downregulation of the MYC pathway. (**E**) Volcano plots showing log_2_FC of gene expression following decitabine treatment for each PDX model. Genes with a P adj value lower than 0.05 are represented in blue (DESeq2). Genes that are differentially methylated and expressed after decitabine treatment in all PDX models are annotated in red. MYC is annotated in green. (**F**) Integrated analysis of differentially expressed genes (log_2_FC) and differentially methylated CpGs (Δβ) in decitabine- versus vehicle-treated PDX models.

To study the downstream effects of decitabine therapy, mice from each of the five T-LBL PDX models received one treatment cycle of decitabine. Treatment commenced when 50% of the leukocytes were hCD45^+^. Mice were sacrificed one week post-treatment start and hCD45^+^ leukemic blasts were collected from the spleen (PDX1-4) or bone marrow (PDX5), depending on their location, for DNA methylation profiling and RNA sequencing (Figure 2A). Additionally, in these mice, an effect of one treatment cycle of decitabine was observed when comparing the spleen size and spleen weight of treated versus control mice (Supplementary Figure S4). Since RNA samples of PDX2 were sequenced in a different batch several years before the other PDX samples to find preliminary data, PDX2 samples were excluded from RNA sequencing analysis to avoid batch effect.

Differential methylation analysis showed a decrease in global DNA methylation after decitabine treatment. However, similar to what has been observed in a T-ALL context, the CpG sites determining the CIMP subtype showed little change in methylation profile after decitabine treatment in our T-LBL models (Figure 2B) [5]. Nevertheless, treatment with decitabine decreased the EpiTOC age in all T-LBL PDX models, reflecting the genome-wide hypomethylation of CpG sites included in the EpiTOC age model (Figure 2C).

Compared to the decitabine-induced widely hypomethylated DNA methylation profile, the effect on the transcriptome was less pronounced (Table 1). To further analyze the mechanisms of action of decitabine in T-LBL, a preranked gene set enrichment analysis (GSEA) was performed on the quantified genes after decitabine treatment in all PDX models. This revealed that decitabine induced overall downregulation of MYC target genes (Figure 2D). RNA sequencing data (Supplementary File S2) also revealed a decreased MYC level in all PDX models (Figure 2E, Supplementary Figure S5). However, the downregulation in PDX4 and PDX5 was less pronounced, which may explain the more limited effects of decitabine in these models. Overall, these findings suggest that downregulation of the MYC pathway plays an important role in the effective treatment of T-LBL with decitabine.

An integrated DNA methylation and gene expression analysis showed that hypomethylation induced by decitabine results in increased as well as decreased expression of genes (Figure 2F, Supplementary File S2). The number of overlapping differentially methylated CpGs and differentially expressed genes varied between the different PDX models (Table 1). A very high proportion of the annotated genes was hypomethylated after decitabine treatment. The majority of differently expressed genes were also differently methylated, but most hypomethylated events did not affect gene expression. Focused analysis of genes that were commonly hypomethylated (Δβ < −0.2) and differentially expressed (*p* < 0.05) after decitabine treatment in all PDX models (PDX2 excluded) showed increased expression of only eight genes (*CMTM6, CXXC5, MYO1F*, *NFE2*, *NOSIP*, *RGS9*, *SUCNR1* and *TPM4)* and decreased expression of one gene (*DNTT*) (Table 2, Figure 2E, Supplementary Figure S6). In three out of four PDX models, *MTUS1*, *BIN1*, *CR1*, *CTAG2*, *CTHRC1*, *EPAS1*, *MCAM*, *MX1*, *PCDH10*, *S100A4*, *S100P* and *TPO* were also hypomethylated with increased expression (Supplementary File S2). Of note, re-expression of these genes was already reported as a key player in the anti-cancer effects of decitabine treatment in other different cancers [25,26,27,28,29,30,31,32,33,34,35,36]. We also observed re-expression of *CCND2*, *CD82* and *IGF1R* in three out of four models, which was already described after azacytidine treatment in several cancers [37,38,39,40]. Re-expression of these genes could contribute to the general anti-cancer effects of DNA hypomethylating agents.

### 3.3. Higher Sensitivity to Decitabine Is Mediated by Downregulation of Genes Involved in the Cell Cycle and DNA Replication

As some patients appeared to have more benefit from being treated with decitabine compared to others, we divided the PDX models into a more (PDX1, PDX2 and PDX3) and less (PDX4 and PDX5) decitabine-sensitive subgroup, based on the benefit in living days and drop in hCD45^+^ cells in the peripheral blood after decitabine treatment. We here explained this difference in sensitivity by comparing the downstream effects of the two groups and determined a gene signature linked to high decitabine sensitivity.

The integrated DNA methylation and gene expression analysis showed evident differences after decitabine treatment between the more sensitive and less sensitive groups (Table 3, Supplementary File S2). Since RNA samples of PDX2 were sequenced in a different batch several years before the other PDX samples to find preliminary data, PDX2 samples were excluded from RNA sequencing analysis to avoid batch effect. Although the number of differentially methylated CpGs is similar in both subgroups, the number of differentially expressed genes is higher in the more sensitive group compared to the less sensitive group (Table 3).

**Figure 3:**
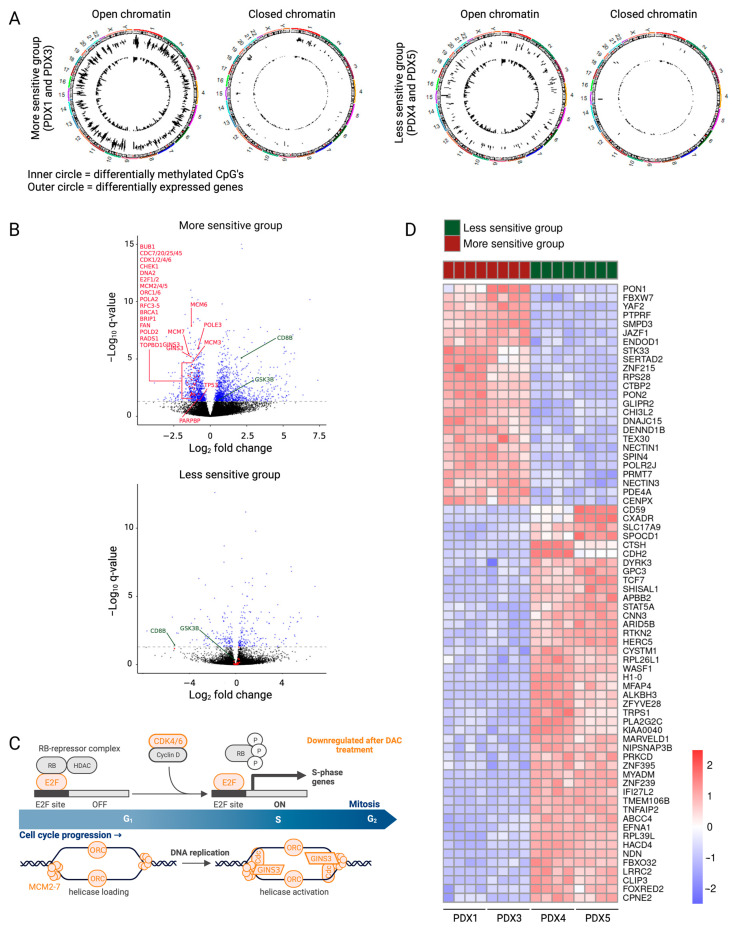
Downstream effects of decitabine explain the differences between the more and less sensitive subgroups. (**A**) Circle plots showing hypomethylated CpGs (inner circle) and differentially expressed genes (outer circle) in the open regions and closed regions of the chromosome for the more sensitive and the less sensitive groups. (**B**) Volcano plots showing log_2_FC of gene expression following treatment with decitabine. Genes with a *p* adj value lower than 0.05 are represented in blue (Deseq2). Differentially methylated and expressed genes involved in the cell cycle, DNA replication and DNA damage are featured in red. Some differentially methylated and expressed genes involved in the activation of the immune system are featured in green. (**C**) Gene set analysis showed downregulation of multiple genes involved in the cell cycle (in orange) in the more sensitive subgroup. Figure created with BioRender.com. (**D**) Heatmap showing the top 70 differentially expressed genes (IFCI > 1 and *p* adj < 0.05) between the more sensitive and less sensitive groups.

To check whether decitabine can influence the expression of genes located in both active and inactive chromatin regions, we made use of publicly available T-ALL HiC data to generate a T-ALL specific chromatin organization architecture. Next, this reference was used to map our differentially methylated CpGs and differently expressed genes in sensitive and less sensitive PDX samples. The differentially methylated CpG sites in both groups mapped to open and closed chromatin regions at a similar proportion as the total number of analyzed CpGs on the Infinium EPIC methylation array (open chromatin location: 82% in array, 80% of DM-CpG in sensitive and 83% of DM-CpG in less sensitive subgroup). However, the differentially expressed genes in both sensitive and less sensitive PDX showed an increased proportion of genes located in an open chromatin region compared with all RNA sequenced genes (open chromatin location: 74% of all RNA sequenced genes, 90% of DEG in sensitive and 94% of DEG in less sensitive PDX) (Figure 3A, Table 4), indicating that decitabine predominantly mediates functional expression changes of genes located in the active chromatin regions. Thus, the chromatin organization of the genes responsible for the anti-lymphoma effects could influence the sensitivity towards decitabine.

Indeed, gene set analysis of the differentially methylated and expressed genes in the more sensitive versus less sensitive group explained the differences in anti-lymphoma effects of decitabine treatment. In the more sensitive group, decitabine treatment resulted in downregulation of genes included in the cell cycle and DNA replication (*BUB1, CDC7/20/25/45*, *CDK1/2/4*, *CHEK1*, *DNA2*, *E2F1/2*, *MCM2-7*, *ORC1/6*, *POLA2*, *RFC3-5*, *TP53*) and DNA repair (*BRCA1*, *BRIP1*, *FAN*, *PARPBP*, *POLD2*, *POLE3*, *RADS1*, *TOPBD1*) (Figure 3B). Downregulation of these pathways induces replicative stress and DNA damage build-up, causing apoptosis. Enrichment analysis of differentially expressed genes on each PDX model separately revealed downregulation of even more genes included in the cell cycle (*CDK6*, *GINS3*, *PA2G4*) in the more sensitive group (Figure 3B,C, Supplementary File S2). Furthermore, the gene set analysis of the differentially methylated and expressed genes in the more sensitive subgroup showed upregulation of multiple gene sets involved in the intrinsic activation of the immune system, even though the treatment was performed in immunodeficient mice. The innate immune system and the adaptive immune response (*CD8B*, *GSK3B*, IFNγ signaling (Supplementary Figure S7)) were upregulated after decitabine treatment in the more sensitive group (Figure 3B).

Since some T-LBL PDX models have more benefit from being treated with decitabine, finding possible biomarkers for higher sensitivity towards decitabine would be of clinical interest. Although the CIMP classification has been shown to serve as a predictor for prognosis [5,43], we see no correlation between the percentage of methylated CIMP CpG sites and the sensitivity towards decitabine. Additionally, decitabine treatment does not affect the CIMP classification of our T-LBL samples (Figure 2B). Since neither the CIMP status nor the EpiTOC age can be used as a biomarker for sensitivity towards decitabine, we here suggest a new gene signature using the top 70 differentially expressed genes between the more sensitive and less sensitive group at base line level (IFCI > 1 and *p* adj < 0.05) as a gene signature for high decitabine sensitivity (Figure 3D, Supplementary File S2).

Altogether, these results show that downregulation of the cell cycle, DNA replication and DNA damage mediate a stronger anti-lymphoma effect in the more sensitive T-LBL subgroup. These different downstream effects can be explained by the activity of decitabine in the open versus closed chromatin regions. However, further studies are required to validate the gene signature as a determinant for high decitabine sensitivity.

## 4. Discussion

In this study, we investigated decitabine as a promising strategy for the treatment of T-LBL. Decitabine has previously been reported to have anti-leukemic properties in other hematological cancers, such as T-ALL and AML [5,6]. Here, we demonstrate that decitabine also has anti-lymphoma effects in several different T-LBL PDX models. Although the TAL/LMO subgroup has been reported to be a T-ALL non-responder to azacytidine [11], another DNA hypomethylating agent, we were able to show the anti-lymphoma effects of decitabine in all of the tested T-LBL subgroups, including the TAL/LMO subgroup (PDX5).

Of note, this study was performed in second generation PDX mice to minimize DNA methylation changes between the patient and the studied model. It was previously reported that the methylation of 97.5% of promotors is preserved between T-ALL patients and their PDX models [18]. Moreover, during the propagation of PDX models, only 1% of CpGs changes methylation level significantly [44]. These studies suggest that the DNA methylome stays fairly preserved between patients and different generations of PDX models.

Although the therapeutic effects of azacytidine and decitabine are undisputed, one of the limitations is that both epidrugs induce lymphoid depletion during treatment. However, this toxicity is relatively low and reversible. Therefore, low doses of decitabine and azacytidine can still be administered with clinically relevant effects [45]. Recently, the new DNMT1-selective inhibitor GSK3685032 has shown improved tolerability and efficacy in AML [46]. Since GSK3685032 only inhibits DNMT1, in contrast to decitabine and azacytidine which also inhibit DNMT3A and DNMT3B, this new inhibitor induces less toxicity to normal blood cells and is worthy of further investigation.

Overall, the genome-wide hypomethylated DNA profile after decitabine treatment had less effect on the transcriptome of the cells. This is in line with several studies that show that decitabine has multiple secondary effects that might induce the changes in gene expression [5,47,48,49]. However, when looking into the downstream effects of decitabine, we did observe downregulation of the oncogenic MYC pathway, contributing to the overall anti-lymphoma effect in T-LBL. Likewise, global downregulation of MYC target genes has been observed after treatment with decitabine in T-ALL, AML and Burkitt lymphoma [5,50,51]. These results suggest that mainly targeting the previously described interplay between the DNA methylating machinery and the MYC-driven tumor maintenance [52] causes the anti-cancer effects of decitabine in T-ALL and T-LBL. Based on research in other hematological malignancies, the anti-cancer effects of decitabine can be enhanced by combination therapy with venetoclax (ABT-199), a BCL-2 inhibitor [53,54]. BCL-2 is an anti-apoptotic protein that has gained therapeutic interest in T-ALL/T-LBL over the last years [55,56]. One case report in a relapsed T-ALL patient and one in a relapsed T-LBL patient showed promising responses after combination therapy of decitabine and venetoclax [57,58]. Further studies to evaluate decitabine in combination with BCL-2 inhibitors for the treatment of relapsed/refractory T-ALL/T-LBL are necessary to provide additional insights into the efficacy of this combination.

**Figure 4:**
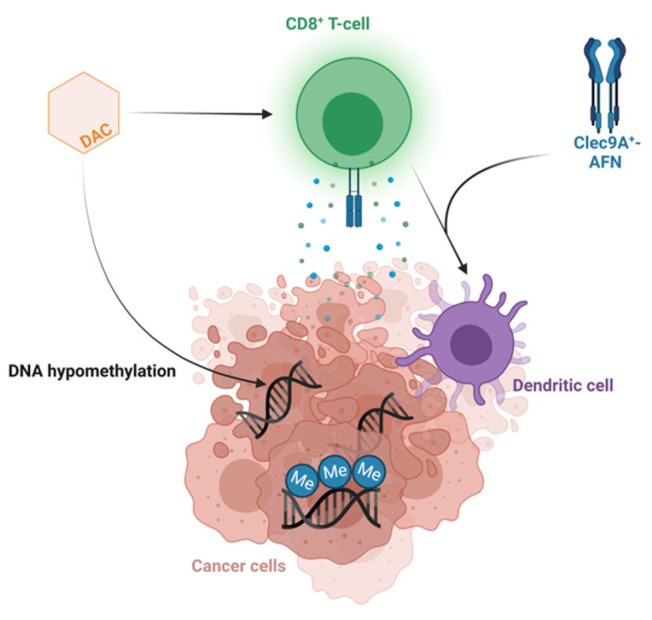
Combining decitabine and Clec9A^+^-AFN treatment as a promising therapeutic strategy. Schematic overview of the hypothesized mechanism of action of the combination of decitabine and Clec9A^+^-AFN treatment. Figure created with BioRender.com.

Some T-LBL patient models derived greater benefit from decitabine therapy compared to others. The more sensitive T-LBL subgroup showed additional downregulation of multiple genes involved in the cell cycle, DNA replication and DNA repair after treatment with decitabine, providing an explanation for the higher anti-lymphoma effect of decitabine in this subgroup. Moreover, multiple gene sets involved in the activation of the immune system were upregulated after decitabine therapy in the more sensitive T-LBL subgroup. We observed increased IFNγ signaling, as previously reported in T-ALL PDX models after treatment with decitabine [5], and an increased CD8 level after decitabine therapy in the more sensitive T-LBL subgroup, in line with the previously reported increased activation and cytolytic activity of CD8^+^ T-cells after decitabine in colorectal cancers [59]. This suggests indirect anti-cancer effects of decitabine treatment induced by IFN-mediated activation of the immune system, demanding antigen presentation by dendritic cells and priming of cytotoxic CD8^+^ T-cells. Therefore, confirming the activation of the immune system in an immunocompetent model can serve as a rationale for combining decitabine with immunotherapy. For instance, looking into potential synergism between decitabine and Clec9A^+^-AFN, activity-on-target interferons inducing immune-driven antitumor effects by activating Clec9A^+^ dendritic cells in T-ALL [60] represents an interesting therapeutic strategy worthy of further investigation in the high-sensitivity subgroup for both T-LBL and T-ALL (Figure 4).

Interestingly, genes located in the open chromatin region clearly make up the bulk of the differentially expressed genes after treatment with decitabine. Since the number of differentially expressed genes is higher in the more sensitive group, this indicates that the genes responsible for the increased anti-lymphoma effect are in open chromatin regions in the more sensitive models. Investigating this in further detail in future studies may be able to better explain the differences between downstream effects and, therefore, sensitivity levels.

Given that there is a less sensitive T-LBL subgroup that might derive greater benefit from other treatment schedules or combination therapies, finding a biomarker for drug sensitivity is of interest. Since the CIMP subtype is not a good biomarker for sensitivity towards decitabine, we here suggested a gene signature that can act as a determinant for high decitabine sensitivity. This gene signature includes the T-ALL/T-LBL oncogenes *TCF7*, *CD59*, *ARID5B* and *STAT5A* which are poorly expressed and *FBXW7*, which is highly expressed in the more sensitive group [61,62,63,64,65,66]. Of note, a high level of *STAT5A* has already shown a correlation with resistance to epidrugs [67]. Additionally, high expression of *RPS28*, *CTBP2*, *JAZF1*, *TCF7* and *NECTIN1* and low expression of *HERC5* and *CTSH* in the more sensitive models have previously been shown to influence drug sensitivity [68,69,70,71,72,73]. Of note, a low methylation level of *JAZF1* has been correlated with a stronger effect of decitabine in endometriosis [71]. However, as the size of our subgroups is limited, further validation of this gene signature with additional and complementary techniques is required in future studies.

## 5. Conclusions

In conclusion, we provide the preclinical evidence that is required for the inclusion of decitabine in clinical trials for patients with T-LBL. Approximately 20% of patients with T-LBL will relapse and the prognosis for relapsed T-LBL is extremely poor. Thus, clinical investigation of decitabine in the relapsed setting should be considered to improve outcomes for these patients. Indeed, decitabine is being implemented in the HEM-iSMART trial in an effort to improve outcomes for relapsed/refractory T-LBL patients.

## Figures and Tables

**Table 1:** The number of differently methylated (DM) annotated genes and differently expressed genes after decitabine treatment differs for each PDX model.

	PDX1	PDX3	PDX4	PDX5
Total number of hypomethylated annotated genes (DM-CpG sites)	31,174 (175,219)	36,431 (308,103)	26,265 (106,835)	38,903 (422,137)
Total number of hypermethylated annotated genes (DM-CpG sites)	51 (44)	44 (41)	5 (4)	95 (75)
Total number of annotated genes with increased expression	1067	2728	151	781
Total number of annotated genes with decreased expression	576	2099	26	700
Hypomethylated annotated genes with increased expression	733	2326	112	689
Hypomethylated annotated genes with decreased expresssion	488	1776	21	619

**Table 2:** After decitabine treatment, nine genes were hypomethylated and differentially expressed in all PDX models. For each gene, the corresponding protein function and previously described associations with decitabine treatment are shown. The number and the location of the differentially methylated CpG probes are indicated.

Gene Name	Protein Function	Previous Association with DAC	Differentially Methylated CpG Sites
*CMTM6*	Regulating T cell activation and antitumor responses	/	Gene: 1
*CXXC5*	Transcription factor and epigenetic regulator in a.o. myelopoiesis	/	Gene: 5 Promotor: 6
*DNTT*	DNA polymerase that functions as DNA nucleotidylexotransferase	/	Gene: 2
*MYO1F*	Immune cell motility	/	Gene: 2 Enhancer: 1
*NFE2*	Megakaryocyte production, regulation of HSC self-renewal and T-cell differentiation by preventing NOTCH1 activation [41]	Decitabine reduces NFE2 expression in HEL cells [42]	Promotor: 4
*NOSIP*	Nitric oxide production	/	Gene: 1 Promotor: 3
*RGS9*	Deactivation of G-proteins	/	Gene: 5 Promotor: 1
*SUCNR1*	Hematopoietic progenitor cell development, regulation immune cell responses	/	Gene: 1
*TPM4*	Actin-binding protein involved in the contractile system, maintains cell-cell adhesions	/	Gene: 3 Enhancer: 1 Promotor: 2

**Table 3:** The number of differently methylated (DM) annotated genes and differently expressed genes after decitabine treatment differs between the more sensitive and less sensitive subgroups.

	More Sensitive Subgroup	Less Sensitive Subgroup
Total number of hypomethylated annotated genes (DM-CpG sites)	34,078 (235,386)	35,017 (272,338)
Total number of hypermethylated annotated genes (DM-CpG sites)	11 (8)	3 (2)
Total number of annotated genes with increased expression	1235	181
Total number of annotated genes with decreased expression	959	133
Hypomethylated annotated genes with increased expression	930	149
Hypomethylated annotated genes with decreased expresssion	808	115

**Table 4:** The differentially expressed genes after decitabine treatment show an increased proportion of genes located in an open chromatin region in both subgroups. The proportion of differently methylated (DM) CpG sites and differently expressed genes (DEG) in the open and closed chromatin regions after decitabine treatment is similar in the more sensitive and less sensitive subgroups. The relative risk is calculated as proportion Open (DM-CpG/DEG)/proportion Open (Not DM-CpG/Not DEG).

	Open Chromatin	Closed Chromatin	Relative Risk
Proportion of CpG sites (number of CpG sites on the Methylation Infinium EPICarray)	0.82 (237,233)	0.18 (53,283)	-
Proportion of genes (number of RNA sequenced genes)	0.74 (9213)	0.26 (3161)	-
**More sensitive subgroup**			
Proportion of DM CpG sites (number of CpG sites)	0.80 (74,802)	0.20 (18,385)	0.98
Proportion of DEG (number of genes)	0.90 (860)	0.10 (96)	1.23
**Less sensitive subgroup**			
Proportion of DM CpG sites (number of CpG sites)	0.83 (90,481)	0.17 (18,132)	1.03
Proportion of DEG (number of genes)	0.94 (144)	0.06 (9)	1.27

## Data Availability

All generated data were deposited in NCBI Gene Expression Omnibus (GEO) under accession number GSE218060.

## Supplementary Materials

The following supporting information can be downloaded at: https://www.mdpi.com/article/10.3390/cancers15030647/s1, Figure S1: Correlation between the Epitoc age and the percentage of methylated CIMP CpGs of the PDX models; Figure S2: The effect of decitabine treatment on the level of hCD45+ cells in the peripheral blood is bigger in some PDX models; Figure S3: Follow-up of lymphoid depletion and leukemic burden in PDX models after decitabine treatment. (A) Mouse CD45 (mCD45) levels in the blood, normalized with precision count beads, are shown after one cycle of decitabine treatment in PDX5. Lymphoid depletion is observed during decitabine treatment. Once treatment is finished, the lymphoid population gets restored within five days. PDX5 is representative for all models. (B) Human CD45 (hCD45) levels in the blood are shown after one cycle of decitabine treatment in PDX3. (Left) The percentage of hCD45^+^ cells was measured in the blood. Due to lymphoid depletion caused by decitabine treatment, the hCD45 percentage is higher in the treated mice compared to the control mice during the treatment. (Right) The number of hCD45^+^ cells in 30 μL blood was measured by normalizing against precision count beads to correct for lymphoid depletion caused by decitabine treatment; Figure S4: One treatment cycle of decitabine shows effect on spleen size and spleen weight. Mice were treated for one cycle of decitabine or vehicle once 50% of cells in the peripheral blood were hCD45^+^. At day 8, mice were sacrificed and the spleen was collected. (A) Spleen size and (B) spleen weight ((spleen weight)/(body weight)) are plotted for all PDX models and shows clear treatment effect; Figure S5: Level of *MYC* after decitabine treatment; Figure S6: Normalized counts of the nine hypomethylated and differentially expressed genes in all PDX models after one treatment cycle of decitabine or vehicle; Figure S7: Preranked GSEA of the IFNγ response from the more sensitive subgroup in decitabine versus vehicle controls; File S1: Characterization of PDX models; File S2: RNAseq and DNAme analysis.
