# Supplementary material for: Pre-Clinical Evaluation of the Hypomethylating Agent Decitabine for the Treatment of T-Cell Lymphoblastic Lymphoma
Source: Cancers (Basel). 2023 Jan 20;15(3):647. doi: 10.3390/cancers15030647 (PMC9913791; doi:10.3390/cancers15030647)
Supplement: Supplementary file 1 [file cancers-15-00647-s001.zip › Supplementary Figures.pdf]

Supplementary Figures

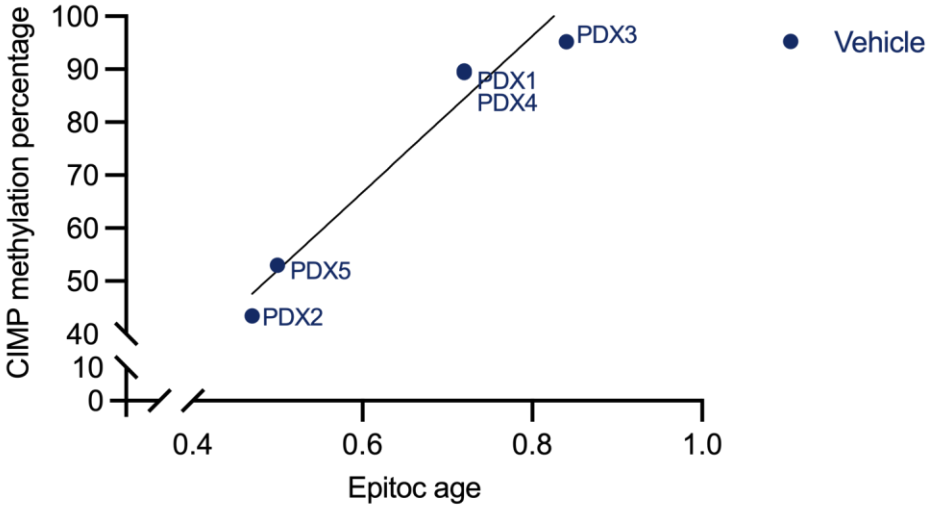

Supplementary Figure S1. Correlation between the Epitoc age and the percentage of methylated CIMP CpGs of the PDX models. Correlation between Epitoc age and the CIMP CpG methylation percentage per PDX model after vehicle treatment.

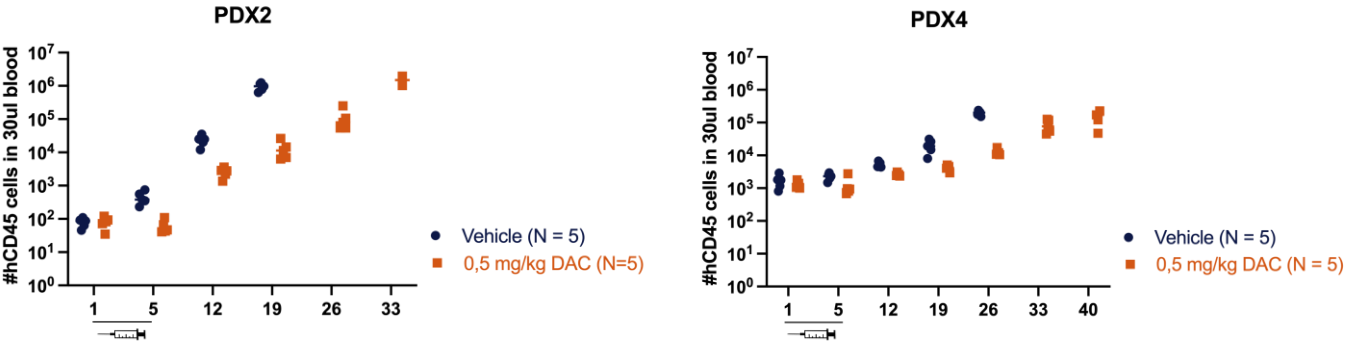

Supplemental Figure S2. The effect of decitabine treatment on the level of hCD45⁺ cells in the peripheral blood is bigger in some PDX models. After 1 treatment cycle, the treatment effect on hCD45⁺ cells in the peripheral blood could be correlated with the treatment effect on survival. PDX models with a higher effect on survival also showed a bigger effect on hCD45⁺ cells, and vice-versa. The number of hCD45⁺ cells in the peripheral blood after 1 treatment cycle was followed-up weekly with flow cytometry. The effect on hCD45⁺ cells is higher in the more sensitive group (left, represented by PDX2) compared to the less sensitive group (right, represented by PDX4).

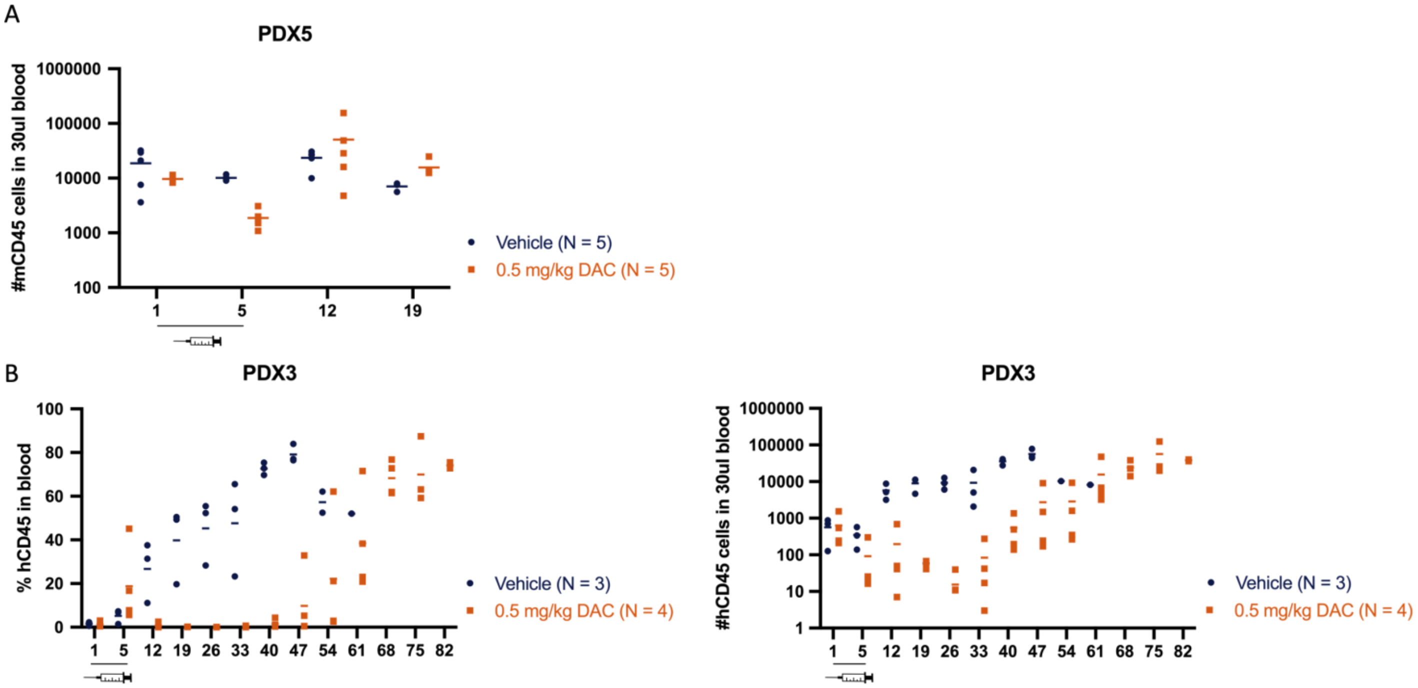

Supplementary Figure S3. Follow-up of lymphoid depletion and leukemic burden in PDX models after decitabine treatment. (A) Mouse CD45 (mCD45) levels in the blood, normalized with precision count beads, are shown after one cycle of decitabine treatment in PDX5. Lymphoid depletion is observed during decitabine treatment. Once treatment is finished, the lymphoid population gets restored within five days. PDX5 is representative for all models. **(B)** Human CD45 (hCD45) levels in the blood are shown after one cycle of decitabine treatment in PDX3. (Left) The percentage of hCD45⁺ cells was measured in the blood. Due to lymphoid depletion caused by decitabine treatment, the hCD45 percentage is higher in the treated mice compared to the control mice during the treatment. (Right) The number of hCD45⁺ cells in 30µl blood was measured by normalizing against precision count beads to correct for lymphoid depletion caused by decitabine treatment. The number of hCD45⁺ cells is lower in the treated mice compared to the control mice during the treatment. PDX3 is representative for all models.

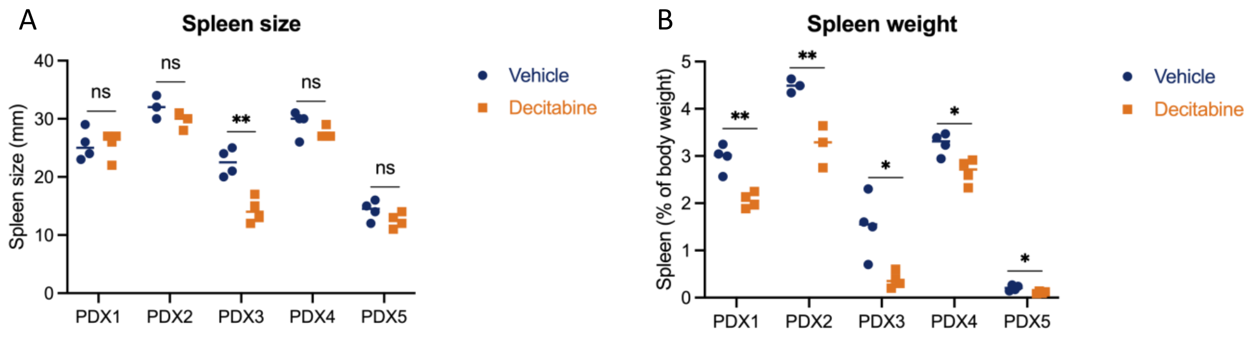

Supplementary Figure S4. One treatment cycle of decitabine shows effect on spleen size and spleen weight. Mice were treated for one cycle of decitabine or vehicle once 50% of cells in the peripheral blood were hCD45⁺. At day 8, mice were sacrificed and the spleen was collected. **(A)** Spleen size and **(B)** spleen weight ((spleen weight)/(body weight)) are plotted for all PDX models and shows clear treatment effect.

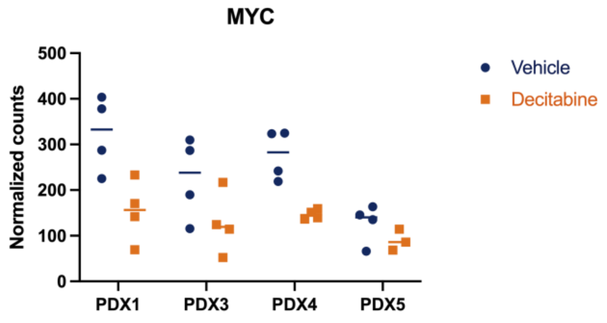

Supplementary Figure S5. Level of *MYC* after decitabine treatment. Normalized counts of *MYC* after one treatment cycle of decitabine or vehicle.

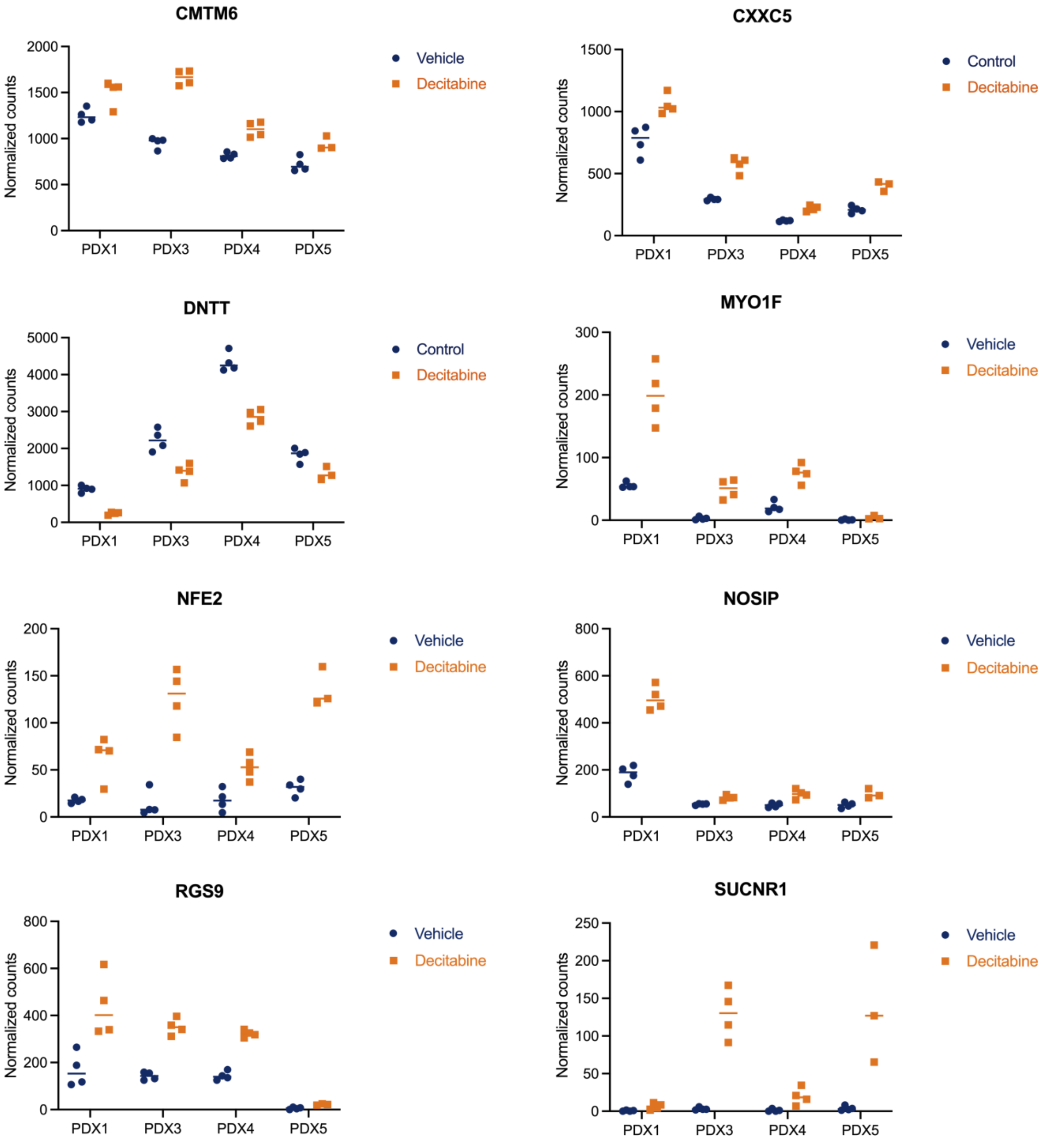

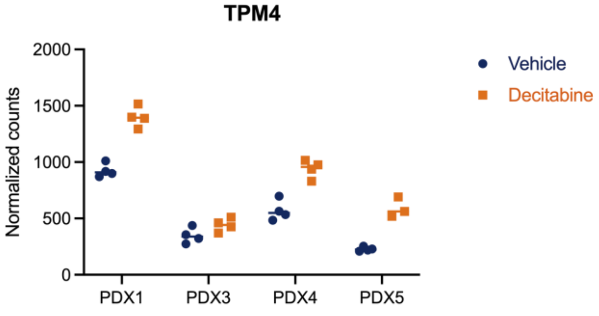

Supplementary Figure S6. Normalized counts of the nine hypomethylated and differentially expressed genes in all PDX models after one treatment cycle of decitabine or vehicle.

PDX1 **PDX3**

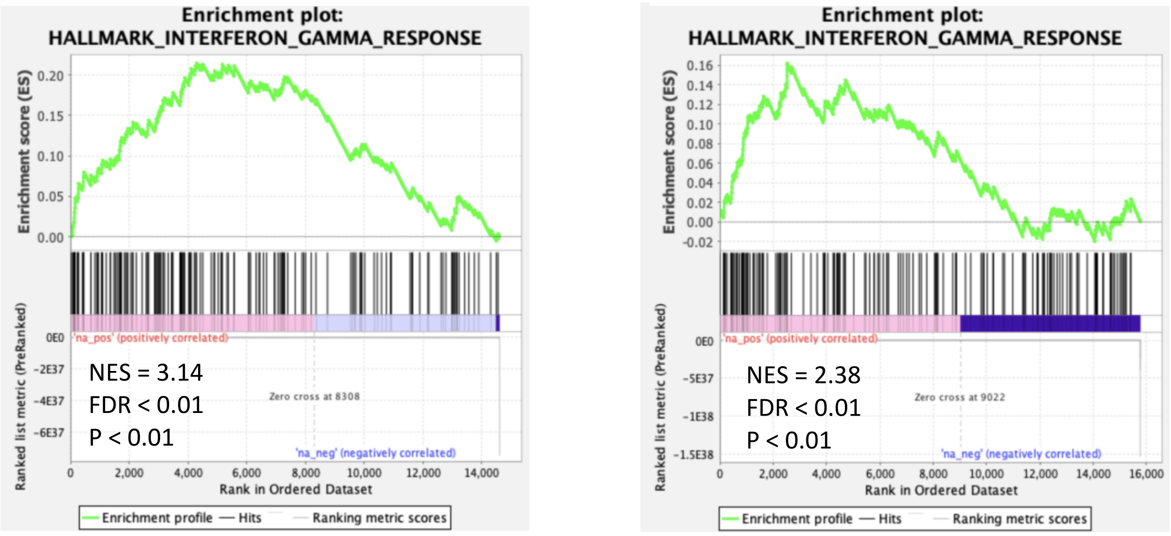

Supplementary Figure S7. Preranked GSEA of the IFNγ response from the more sensitive subgroup in decitabine versus vehicle controls. The IFNγ response is upregulated in both PDX1 and PDX3 after decitabine treatment.
